# Effect of Extraction Solvent/Technique on the Antioxidant Activity of Selected Medicinal Plant Extracts

**DOI:** 10.3390/molecules14062167

**Published:** 2009-06-15

**Authors:** Bushra Sultana, Farooq Anwar, Muhammad Ashraf

**Affiliations:** 1 Department of Chemistry and Biochemistry, University of Agriculture, Faisalabad-38040, Pakistan; 2 Department of Botany, University of Agriculture, Faisalabad-38040, Pakistan

**Keywords:** medicinal plants, extraction effect, total phenolics, total flavonoids, antioxidant activity

## Abstract

Theeffects of four extracting solvents [absolute ethanol, absolute methanol, aqueous ethanol (ethanol: water, 80:20 v/v) and aqueous methanol (methanol: water, 80:20 v/v)] and two extraction techniques (shaking and reflux) on the antioxidant activity of extracts of barks of *Azadirachta indica*, *Acacia nilotica, Eugenia jambolana*, *Terminalia arjuna*, leaves and roots of *Moringa oleifera*, fruit of *Ficus religiosa*, and leaves of *Aloe barbadensis* were investigated. The tested plant materials contained appreciable amounts of total phenolic contents (0.31-16.5 g GAE /100g DW), total flavonoid (2.63-8.66 g CE/100g DW); reducing power at 10 mg/mL extract concentration (1.36-2.91), DPPH**^.^** scavenging capacity (37.2-86.6%), and percent inhibition of linoleic acid (66.0-90.6%). Generally higher extract yields, phenolic contents and plant material antioxidant activity were obtained using aqueous organic solvents, as compared to the respective absolute organic solvents. Although higher extract yields were obtained by the refluxing extraction technique, in general higher amounts of total phenolic contents and better antioxidant activity were found in the extracts prepared using a shaker.

## Introduction

Plant-derived antioxidants, especially, the phenolics have gained considerable importance due to their potential health benefits. Epidemiological studies have shown that consumption of plant foods containing antioxidants is beneficial to health because it down-regulates many degenerative processes and can effectively lower the incidence of cancer and cardio-vascular diseases [1].

Recovery of antioxidant compounds from plant materials is typically accomplished through different extraction techniques taking into account their chemistry and uneven distribution in the plant matrix. For example, soluble phenolics are present in higher concentrations in the outer tissues (epidermal and sub-epidermal layers) of fruits and grains than in the inner tissues (mesocarp and pulp) [2]. Solvent extraction is most frequently used technique for isolation of plant antioxidant compounds. However, the extract yields and resulting antioxidant activities of the plant materials are strongly dependent on the nature of extracting solvent, due to the presence of different antioxidant compounds of varied chemical characteristics and polarities that may or may not be soluble in a particular solvent. Polar solvents are frequently employed for the recovery of polyphenols from a plant matrix. The most suitable of these solvents are (hot or cold) aqueous mixtures containing ethanol, methanol, acetone, and ethyl acetate [3]. Methanol and ethanol have been extensively used to extract antioxidant compounds from various plants and plant-based foods (fruits, vegetables etc.) such as plum, strawberry, pomegranate, broccoli, rosemary, sage, sumac, rice bran, wheat grain and bran, mango seed kernel, citrus peel, and many other fruit peels. Other studies have also demonstrated the efficacy of ethyl acetate to extract phenolic compounds from onion and citrus peel [3,4,5,6]. Bonoli *et al*. [7] reported that maximum phenolic compounds were obtained from barley flour with mixtures of ethanol and acetone. Similarly, aqueous methanol was found to be more effective in recovering highest amounts of phenolic compounds from rice bran [8], and *Moringa oleifera* leaves [9]. Anwar *et al*. [10] extracted antioxidant compounds from various plant materials including rice bran, wheat bran, oat groats and hull, coffee beans, citrus peel and guava leaves using aqueous 80% methanol (methanol: water, 80:20 v/v). 

The medicinal plants selected for the present investigation, which included *Moringa oleifera, Azadirachta indica, Terminalia arjuna, Acacia nilotica, Eugenia jambolana, Aloe barbadensis* etc*.* have long been used in the folk medicine due to their potential health promoting and pharmacological attributes, which are mainly ascribed to the presence of antioxidant constituents such as phenolic acids and flavonoids [9,11,12,13]. It is important to establish appropriate means to evaluate and quantify effective antioxidant principles of medicinally or economically viable plant materials. The present study therefore was conducted with the main objective of investigating the most effective solvent/technique for extracting potent antioxidant compounds, especially phenolics from different parts of selected medicinal plants native to Pakistan. 

## Results and Discussion

### Effects of extracting solvent/technique on the extracts yields from different medicinal plant materials

Amounts (g/100g of dried plant material) of the antioxidant extract determined for different medicinal plant materials, using four different solvents (absolute methanol and aqueous methanol (methanol: water, 80:20 v/v); absolute ethanol and aqueous ethanol, (ethanol: water, 80:20 v/v) and two extracting techniques: shaker and reflux are shown in Table 1. 

**Table 1:** Effects of extracting solvent/technique on the extract yield (g/100 g of DW) of medicinal plant materials.

Medicinal plant organs	Extraction by shaker			
	Absolute methanol	Aqueous (80%) methanol	Absolute ethanol	Aqueous (80%) ethanol
*Moringa oleifera* leaves	9.61 ± 0.39_d_^c^	17.9 ± 0.18_c_^a^	8.94 ± 0.27_d_^c^	12.6 ± 0.51_d_^b^
*Moringa oleifera* root	3.24 ± 0.14_e_^b^	6.65 ± 0.19_e_^a^	2.23 ± 0.12_e_^b^	3.63 ± 0.26_e_^b^
*Eugenia jambolana* bark	19.2 ± 0.38_b_^a^	14.1 ± 0.56_d_^b^	2.81 ± 0.39_e_^c^	13.5 ± 0.40_d_^b^
*Acacia nilotica* bark	23.4 ± 0.47_a_^b^	31.6 ± 0.95_a_^a^	13.1 ± 0.52_b_^c^	15.7 ± 0.32_c_^c^
*Azadirachta indica* bark	10.7 ± 0.22_d_^c^	13.8 ± 0.55_d_^c^	37.2 ± 0.74_a_^a^	25.0 ± 0.53_b_^b^
*Terminalia arjuna* bark	22.5 ± 0.67_a_^b^	23.3 ± 0.45_b_^b^	34.5 ± 0.44_a_^a^	37.2 ± 0.46_a_^a^
*Ficus religiosa* fruit	18.9 ± 0.76_b_^b^	26.4 ± 0.52_b_^a^	16.9 ± 0.67_b_^b^	19.7 ± 0.39_c_^b^
*Aloe barbadensis* leaves	15.6 ± 0.62_c_^b^	17.8 ± 0.36_c_^a^	10.8 ± 0.43_d_^c^	15.2 ± 0.68_c_^b^
	**Extraction by reflux**			
*Moringa oleifera* leaves	16.6 ± 0.33_d_^b^	21.1 ± 0.84_c_^a^	12.2 ± 0.37_c_^c^	17.2 ± 0.35_c_^b^
*Moringa oleifera* root	5.12 ± 0.21_e_^bc^	8.97 ± 0.36_d_^a^	4.86 ± 0.21_d_^c^	6.27 ± 0.26_d_^b^
*Eugenia jambolana* bark	25.6 ± 0.51_b_^a^	16.9 ± 0.33_c_^c^	15.3 ± 0.37_bc_^c^	19.5 ± 0.46_c_^b^
*Acacia nilotica* bark	26.2 ± 0.78_b_^b^	32.8 ± 0.65_a_^a^	18.2 ± 0.55_b_^c^	20.2 ± 0.61_c_^c^
*Azadirachta indica* bark	14.2 ± 0.29_d_^c^	17.8 ± 0.53_c_^c^	42.4 ± 0.64_a_^a^	31.9 ± 0.63_b_^b^
*Terminalia arjuna* bark	28.6 ± 0.46_a_^b^	24.9 ± 0.49_b_^b^	40.7 ± 0.86_a_^a^	46.6 ± 083_a_^a^
*Ficus religiosa* fruit	21.3 ± 0.64_c_^c^	29.2 ± 0.88_a_^a^	19.5 ± 0.58_b_^c^	22.8 ± 0.91_c_^c^
*Aloe barbadensis* leaves	17.5 ± 0.73_d_^b^	20.3 ± 0.41_c_^a^	13.2 ± 0.52_c_^c^	18.1 ± 0.72_c_^b^

Values (mean ± SD) are average of three samples of each medicinal plant material, analyzed individually in triplicate (*n* = 1x3 x 3), (*P* < 0.05); DW= dry weight; Superscript letters within the same row indicate significant (*P*< 0.05) differences of means within the extracting solvent; Subscript letters within the same column indicate significant (*P*< 0.05) differences of means within the plant materials.

Our findings are in agreement with previous investigation of Chatha *et al*. [8], who reported that maximum extract yield (g/100g) from rice bran was obtained with aqueous methanol.

The differences in the extract yields from the tested plant materials in the present analysis might be ascribed to the different availability of extractable components, resulting from the varied chemical composition of plants [14]. The amount of the antioxidant components that can be extracted from a plant material is mainly affected by the vigor of the extraction procedure, which may probably vary from sample to sample. Amongst other contributing factors, efficiency of the extracting solvent to dissolve endogenous compounds might also be very important [9,15]. 

For the effectiveness of extracting technique, the results showed that yields of the extract were better when extraction was done under reflux, regardless of the plant material and solvent used. This indicates that hot solvent systems under reflux state are more efficient for the recovery of antioxidant components, thus offering higher extract yields. This is in agreement with the findings of Shon *et al*. [16] who investigated that methanol and hot water are more efficient to extract antioxidant compounds from *Phellinus baumii*. 

### Effects of extracting solvent/technique on the total phenolic contents of different plant materials

Total phenolic contents (TPC) of different plant materials, using four solvent systems: absolute and aqueous methanol and absolute and aqueous ethanol and two extracting techniques (shaker and reflux) are presented in Table 2. Among the different medicinal plant materials, aqueous ethanolic extract of *Acacia nilotica* bark offered the highest TPC (16.5 g GAE/100g of DW), followed by aqueous ethanolic extract (aq. EE) of *Terminalia arjuna* bark (12.8 %), aq. ME of *Moringa oleifera* leaves (12.2%), aq. EE of *Azadirachta indica* bark (12.0%), aq. ME of *Aloe barbadensis* leaves (10.3%), aq. EE of *Eugenia jambolana* bark (9.03%), aq. ME of *Ficus religiosa* fruit (5.34%), and aq. ME of *Moringa oleifera* roots (0.31%). 

**Table 2:** Effects of extracting solvent/technique on the total phenolic contents (GAE g/100 g of DW) of medicinal plants materials.

Medicinal plant organs	Extraction by shaker			
	Absolute methanol	Aqueous (80%) methanol	Absolute ethanol	Aqueous (80%) ethanol
*Moringa oleifera* leaves	10.3 ± 0.41_ab_^ab^	12.2 ± 0.28_a_^a^	9.72 ± 0.21_a_^b^	11.6 ± 0.21_b_^ab^
*Moringa oleifera* root	0.22 ± 0.07_d_^b^	0.31± 0.06_e_^a^	0.14 ± 0.01_e_^c^	0.27 ± 0.08_f_^ab^
*Eugenia jambolana* bark	10.1 ± 0.39_ab_^a^	8.30 ± 0.49_b_^b^	8.12 ± 0.35_b_^b^	9.03 ± 0.45_c_^ab^
*Acacia nilotica* bark	12.7 ± 0.28_a_^b^	11.2 ± 0.33_ab_^b^	11.2 ± 0.31_a_^b^	16.5 ± 0.66_a_^a^
*Azadirachta indica* bark	11.1 ± 0.66_ab_^ab^	9.34 ± 0.37_ab_^ab^	8.48 ± 0.26_b_^b^	12.0 ± 0.36_b_^a^
*Terminalia arjuna* bark	12.2 ± 0.57_a_^a^	7.80 ± 0.39_c_^b^	10.2 ± 0.39_a_^ab^	12.8 ± 0.26_b_^a^
*Ficus religiosa* Fruit	3.13 ± 0.19_c_^ab^	5.34 ± 0.36_d_^a^	2.67 ± 0.16_d_^b^	4.11 ± 0.18_e_^ab^
*Aloe barbadensis* leaves	8.25 ± 0.28_b_^ab^	10.3 ± 0.28_ab_^a^	6.53 ± 0.38_c_^b^	7.93 ± 0.31_d_^ab^
	**Extraction by reflux**			
*Moringa oleifera* leaves	9.63 ± 0.28_b_^ab^	10.7 ± 0.31_a_^a^	6.16 ± 0.26_c_^b^	8.21 ± 0.36_c_^ab^
*Moringa oleifera* root	0.17 ± 0.02_e_^c^	0.27 ± 0.04_e_^a^	0.12 ± 0.03_e_^d^	0.23 ± 0.06_f_^b^
*Eugenia jambolana* bark	8.91 ± 0.39_c_^a^	8.14 ± 0.33_b_^a^	7.94 ± 0.31_b_^a^	8.64 ± 0.27_c_^a^
*Acacia nilotica* bark	12.22 ± 0.21_a_^ab^	10.7 ± 0.24_a_^b^	10.8 ± 0.28_a_^b^	14.6 ± 0.29_a_^a^
*Azadirachta indica* bark	9.72 ± 0.33_b_^a^	7.91 ± 0.39_b_^b^	7.23 ± 0.23_b_^b^	10.8 ± 0.38_b_^a^
*Terminalia arjuna* bark	11.63 ± 0.29_ab_^a^	6.25 ± 0.30_c_^b^	9.67 ± 0.38_a_^ab^	11.9 ± 0.46_b_^a^
*Ficus religiosa* Fruit	2.12 ± 0.09_d_^b^	4.93 ± 0.28_d_^a^	2.26 ± 0.10_d_^b^	4.13 ± 0.21_e_^a^
*Aloe barbadensis* leaves	7.29 ± 0.27_c_^b^	9.24 ± 0.26_ab_^a^	6.44 ± 0.29_c_^b^	6.94 ± 0.27_d_^f^

Values (mean ± SD) are average of three samples of each medicinal plant material, analyzed individually in triplicate (*n* = 1x3 x 3), (*P* < 0.05); DW= dry weight; Superscript letters within the same row indicate significant (*P*< 0.05) differences of means within the extracting solvent; Subscript letters within the same column indicate significant (*P*< 0.05) differences of means within the plant materials.

Results of the present study showed that among all the solvent extracts; the aqueous methanol and aqueous ethanol extracts had the highest TPC. This may be due to the fact that phenolics are often extracted in higher amounts in more polar solvents such as aqueous methanol/ethanol as compared with absolute methanol/ethanol [9,10,15]. 

The determined amounts of total phenolics (TP) from the tree barks investigated in the present study were lower than that reported for *Acacia confusa* bark [17]. Except for *Eugenia jambolana*, the barks of the other three plants offered greater amount of total phenolics than those of pine bark (11.4 g GCE/100g DW) [18]. TPC of *Moringa oleifera* leaves investigated in the present analysis are in agreement with previous reports [19]. The amount of TP of *Moringa oleifera* roots were found to be lower than those of Chinese herbal roots of kudzu vine (1.37 g GAE/100g) and dahurian (1.2 g GAE/100g) [20]. The levels of TP determined in the present analysis of *Ficus religiosa* fruit were found to be lower than those reported in *Ficus microcarpa* fruit (17.9 g GAE/100g) [21].

In contrast to the trends noted for extraction yields, the TPC of all medicinal plant materials extracted using the reflux technique decreased, regardless of the nature of the extracting solvent used. The decrease in the amounts of TP of these plant material extracts, prepared under reflux might have been due to the thermal decomposition of some phenolic antioxidants at the higher temperatures used for reflux extraction. 

It has been reported that thermal processing conditions might result in the loss of natural antioxidants because heat may accelerate their oxidation and other degenerative reactions. Thus, heating temperature is of much consideration during processing. An accelerated shelf-life test at 80 °C for 4 days resulted in 20-40% decrease of the antioxidant activity of the apple juice [22]. Cheng *et al*. [23] reported that antioxidant activity of wheat bran decreased up to 61% by heating at 100 °C for 9 days. On the other hand, Dutra *et al*. [24] reported that among different extraction techniques (reflux, maceration, ultrasound, heating plate), extraction made under reflux using ethanol/water (70:30, v/v) offered the highest polyphenol levels in Vogel seeds. This might be attributed to an effective extraction under reflux conditions leading to higher release of some bound phenolics [2]. 

### Effects of extracting solvent/technique on the total flavonoids of different plant materials

Total flavonoid contents (TFC) of various plant materials, extracted with four different solvent systems, using shaker and reflux extracting techniques, are given in Table 3. TFC were determined as catechin equivalents (CE). Among medicinal plant materials, aq. ME of *Moringa oleifera* leaves offered the highest TFC (8.66 g CE/100 g of DW) followed by aq. EE of *Acacia nilotica* bark (4.93), aq. ME of *Aloe barbadensis* leaves (2.28), aq. ME of *Ficus religiosa* fruit (3.77), aq. EE of *Terminalia arjuna* bark (3.49), aq. EE of *Azadirachta indica* bark (3.14), aq. ME of *Moringa oleifera* root(2.94), and ab. ME of *Eugenia jambolana* bark (2.63). Amount of TF in all the medicinal plant extracts generally decreased when reflux technique employed for their preparation. However, TFC of *Aloe barbadensis* leaves increased from 4.28 to 4.66 g CE/100 g of DW, when extracted with aqueous methanol using the reflux technique. *Ficus religiosa* fruits also contained higher TFC using the reflux technique with absolute and aqueous ethanol. TFC (1.47-3.77g/100g) of *Ficus religiosa* fruit in our analysis were found to be higher than that reported for *Ficus microcarpa* fruit (0.6 g/100 g dry weight) [21]. On the other hand, TFC in *Terminalia arjuna* bark (1.52-3.49 g/100g) determined in our work were lower than those (5.70 g/100 g dry weight) investigated by Dwivedi [12].

**Table 3:** Effects of extracting solvent/technique on the total flavonoid contents (CE g/100 g of DW) of medicinal plants materials.

Medicinal plant organs	Extraction by shaker			
	Absolute methanol	Aqueous (80%) methanol	Absolute ethanol	Aqueous (80%) ethanol
*Moringa oleifera* leaves	6.06 ± 0.12_a_^b^	8.66 ± 0.21_a_^a^	5.33 ± 0.13_a_^b^	6.21 ± 0.11_a_^b^
*Moringa oleifera* root	1.68 ± 0.06_d_^b^	2.94 ± 0.08_c_^a^	1.22 ± 0.04_e_^b^	1.59 ± 0.03_d_^b^
*Eugenia jambolana* bark	2.63 ± 0.04_c_^a^	1.72 ± 0.07_d_^ab^	1.68 ± 0.06_d_^b^	2.10 ± 0.06_cd_^ab^
*Acacia nilotica* bark	4.86 ± 0.09_b_^a^	3.21 ± 0.12_bc_^b^	3.15 ± 0.14_b_^b^	4.93 ± 0.15_b_^a^
*Azadirachta indica* bark	2.93 ± 0.10_c_^a^	3.31 ± 0.16_bc_^a^	2.68 ± 0.12_c_^a^	3.14 ± 0.09_c_^a^
*Terminalia arjuna* bark	3.01 ± 0.13_c_^ab^	2.13 ± 0.13_c_^b^	2.64 ± 0.09_c_^ab^	3.49 ± 0.11_c_^a^
*Ficus religiosa* fruit	2.16 ± 0.08_c_^b^	3.77 ±0.10_bc_^a^	1.28 ± 0.04_e_^c^	2.03 ± 0.06_cd_^b^
*Aloe barbadensis* leaves	2.91 ± 0.16_c_^b^	4.28 ± 0.17_b_^a^	1.68 ± 0.02_d_^c^	2.96 ± 0.04_c_^b^
	**Extraction by reflux**			
*Moringa oleifera* leaves	5.90 ± 0.16_a_^b^	7.29 ± 0.18_a_^a^	4.19 ± 0.09_a_^c^	5.31 ± 0.19_a_^b^
*Moringa oleifera* root	1.02 ± 0.03_e_^b^	2.86 ± 0.13_c_^a^	0.89 ± 0.07_e_^b^	1.21 ± 0.07_d_^b^
*Eugenia jambolana* bark	1.99 ± 0.06_d_^a^	0.83 ± 0.02_e_^bc^	1.06 ± 0.04_de_^c^	1.55 ± 0.05_d_^b^
*Acacia nilotica* bark	3.92 ± 0.12_b_^a^	2.52 ± 0.06_c_^b^	3.00 ± 0.13_b_^b^	4.19 ± 0.11_b_^a^
*Azadirachta indica* bark	2.16 ± 0.08_d_^b^	2.90 ± 0.04_c_^a^	1.66 ± 0.06_d_^c^	2.99 ± 0.13_c_^a^
*Terminalia arjuna* bark	1.78 ± 0.06_d_^ab^	1.52 ± 0.05_d_^b^	2.11 ± 0.11_c_^ab^	2.63 ± 0.08_c_^a^
*Ficus religiosa* fruit	1.97 ± 0.05_d_^c^	3.56 ± 0.11_bc_^a^	1.47 ± 0.05_d_^c^	2.86 ± 0.12_c_^b^
*Aloe barbadensis* leaves	2.90 ± 0.07_c_^b^	4.66 ± 0.09_b_^a^	1.39 ± 0.07_d_^c^	2.55 ± 0.09_c_^b^

Values (mean ± SD) are average of three samples of each medicinal plant material, analyzed individually in triplicate (*n* = 1x3 x 3), (*P* < 0.05); DW= dry weight; Superscript letters within the same row indicate significant (*P*< 0.05) differences of means within the extracting solvent; Subscript letters within the same column indicate significant (*P*< 0.05) differences of means within the plant materials.

### Effects of extracting solvent/technique on the reducing power of different plant materials

The results showing the effects of extracting solvent/techniques on the reducing potential of extracts of different plant materials at concentration of 10 mg/mL, are shown in Table 4. The reducing power of the medicinal plant extracts increased in a concentration dependent manner (data not shown). The values of absorbance for the tested extract solutions at concentration of 10 mg/mL determined in this assay, ranged from 0.09 to 2.88 and followed the order of effectiveness as: aq. ME of *Moringa oleifera* leaves (2.88)> aq. ME of *Aloe barbadensis* leaves (2.81) > aq. EE of *Acacia nilotica* bark (1.87)> aq. EE of *Azadirachta indica* bark (1.71) > aq. ME of *Terminalia arjuna* bark (1.66) > aq. EE of *Eugenia jambolana* bark (1.60) > aq. ME of *Ficus religisa* fruit (1.36) > aq. ME of *Moringa oleifera* roots (0.14). 

In general, the aqueous organic solvent extracts of the tested plant materials, exhibiting greater TPC, also depicted good reducing power in the present analysis. The reducing potential of antioxidant components is very much associated with their TPC. The plant extracts with higher levels of total phenolics also exhibit greater reducing power [23,9,15]. 

As far as the effects of extraction techniques on the antioxidant activity is concerned, apart from the leaves of *Aloe vera* and fruit of *Ficus religiosa*, reducing powers of all the medicinal plant materials extracts were adversely affected by reflux extracting technique, regardless of the solvent used. However, each material tested retained the same efficacy order as displayed in the case of shaker extraction. 

**Table 4:** Effects of extracting solvent/technique on reducing power (expressed as absorbance values at 700 nm) of different medicinal plant materials.

Medicinal plant organs	Extraction by shaker			
	Absolute methanol	Aqueous (80%) methanol	Absolute ethanol	Aqueous (80%) ethanol
*Moringa oleifera* leaves	2.45 ± 0.05_a_^b^	2.88 ± 0.03_a_^a^	1.53 ± 0.04_a_^c^	2.50 ± 0.06_a_^b^
*Moringa oleifera* root	0.09 ± 0.01_e_^b^	0.14 ± 0.02_c_^a^	0.09 ± 0.01_c_^b^	0.12 ± 0.02_d_^a^
*Eugenia jambolana* bark	1.06 ± 0.04_d_^c^	1.48 ± 0.04_b_^b^	0.98 ± 0.02_b_^c^	1.60 ± 0.03_bc_^a^
*Acacia nilotica* bark	1.68 ± 0.05_c_^ab^	1.52 ± 0.04_b_^ab^	1.45 ± 0.06_a_^b^	1.87 ± 0.05_b_^a^
*Azadirachta indica* bark	1.55 ± 0.03_c_^b^	1.46 ± 0.05_b_^b^	1.05 ± 0.05_b_^c^	1.71 ± 0.03_b_^a^
*Terminalia arjuna* bark	1.26 ± 0.02_cd_^ab^	1.66 ± 0.04_b_^a^	1.12 ± 0.02_b_^b^	1.34 ±0.04_bc_^ab^
*Ficus religiosa* Fruit	1.06 ± 0.04_d_^b^	1.36 ± 0.07_b_^a^	0.92 ± 0.06_b_^b^	0.99 ± 0.07_cd_^b^
*Aloe barbadensis* leaves	2.01 ± 0.03_b_^b^	2.81 ± 0.05_a_^a^	1.56 ± 0.04_a_^c^	2.16 ± 0.04_a_^b^
	**Extraction by reflux**			
*Moringa oleifera* leaves	1.25 ± 0.05_b_^b^	1.78 ± 0.03_b_^a^	0.94 ± 0.04_bc_^c^	0.95 ± 0.04_c_^c^
*Moringa oleifera* root	0.06 ± 0.01_d_^b^	0.13 ± 0.02_d_^a^	0.09 ± 0.03_d_^b^	0.11 ± 0.02_d_^a^
*Eugenia jambolana* bark	0.80 ± 0.04_c_^b^	1.26 ± 0.05_cd_^a^	0.61 ± 0.05_c_^b^	1.39 ± 0.07_bc_^a^
*Acacia nilotica* bark	1.25 ± 0.06_b_^ab^	1.13 ± 0.04_d_^b^	1.05 ± 0.07_bc_^b^	1.62 ± 0.05_b_^a^
*Azadirachta indica* bark	1.16 ± 0.03_b_^b^	1.10 ± 0.07_d_^b^	0.79 ± 0.02_c_^b^	1.56 ± 0.06_b_^a^
*Terminalia arjuna* bark	1.11 ± 0.05_b_^b^	1.46 ± 0.02_c_^a^	0.62 ± 0.03_c_^c^	0.99 ± 0.03_c_^b^
*Ficus religiosa* Fruit	1.13 ± 0.02_b_^b^	1.22 ± 0.05_cd_^a^	1.26 ± 0.06_b_^a^	1.32 ± 0.02_bc_^a^
*Aloe barbadensis* leaves	2.18 ± 0.04_a_^b^	2.96 ± 0.08_a_^a^	1.72 ± 0.04_a_^c^	1.88 ± 0.04_a_^c^

Values (mean ± SD) are average of three samples of each medicinal plant material, analyzed individually in triplicate (*n* = 1x3 x 3), (*P* < 0.05); DW= dry weight; Superscript letters within the same row indicate significant (*P*< 0.05) differences of means within the extracting solvent; Subscript letters within the same column indicate significant (*P*< 0.05) differences of means within the plant materials.

### Effects of extracting solvent/technique on the DPPH. Scavenging activity (% DPPH. remaining) of different plant materials

DPPH^.^ scavenging activity of different plant materials as affected by extracting methods is depicted in Table 5. Absorbance in this assay was recorded at 0.5 to 10 min time intervals from initiation of the reaction. Observed scavenging activity was similar at the beginning of the reaction and changed with increase in the reaction time until it stabilized by the 10^th^ min. Significant (*p*< 0.05) differences of DPPH^. ^scavenging capacities among extracts were observed at 5^th^ minute of the reaction. The DPPH^. ^scavenging ability of the sample extracts was reported as the percent of DPPH^.^ scavenged (% DPPH^.^ scavenging). As expected, a higher percent of DPPH^.^ scavenging is correlated to a higher antioxidant activity [15,20]. 

**Table 5:** Effects of extracting solvent/technique the DPPH^.^ scavenging activity (%) of different medicinal plant materials.

Medicinal plant organs	Extraction by shaker			
	Absolute methanol	Aqueous (80%) methanol	Absolute ethanol	Aqueous (80%) ethanol
*Moringa oleifera* leaves	82.8 ± 1.7_a_^a^	86.3 ± 1.8_a_^a^	70.9 ± 2.2_b_^b^	85.2 ± 1.7_a_^a^
*Moringa oleifera* root	51.4 ± 2.2_cd_^b^	62.9 ± 1.6_b_^a^	56.6 ± 1.2_cd_^ab^	61.8 ± 2.3_c_^a^
*Eugenia jambolana* bark	48.8 ± 1.4_d_^b^	52.7 ± 1.3_bc_^a^	50.4 ± 1.1_d_^ab^	53.9 ± 1.9_d_^a^
*Acacia nilotica* bark	56.3 ± 1.6_c_^b^	60.9 ± 1.4_b_^b^	82.5 ± 1.7_a_^a^	86.6 ± 1.5_a_^a^
*Azadirachta indica* bark	53.2 ± 1.5_cd_^b^	57.4 ± 1.3_b_^ab^	57.3 ± 1.4_cd_^ab^	60.8 ± 1.8_c_^a^
*Terminalia arjuna* bark	37.7 ± 1.3_e_^c^	48.5 ± 1.5_c_^b^	62.9 ± 1.5_c_^a^	67.4 ± 1.4_b_^a^
*Ficus religiosa* fruit	57.2 ± 1.7_c_^b^	63.4 ± 1.1_b_^a^	55.3 ± 1.3_cd_^b^	60.1 ± 1.9_c_^a^
*Aloe barbadensis* leaves	73.7 ± 1.3_b_^b^	80.1 ± 2.3_a_^a^	67.2 ± 1.9_b_^c^	70.7 ± 1.2_b_^b^
	**Extraction by reflux**			
*Moringa oleifera* leaves	81.6 ± 1.9_a_^a^	79.4 ± 1.6_a_^b^	69.2 ± 1.3_b_^c^	80.6 ± 1.8_a_^a^
*Moringa oleifera* root	53.9 ± 1.5_c_^b^	64.8 ± 1.2_b_^a^	58.8 ± 1.6_c_^ab^	62.7 ± 1.9_c_^a^
*Eugenia jambolana* bark	48.1 ± 1.7_c_^b^	51.4 ± 1.4_c_^a^	49.2 ± 1.2_d_^ab^	52.7 ± 1.5_d_^a^
*Acacia nilotica* bark	55.8 ± 1.3_c_^c^	60.7 ± 1.6_b_^b^	81.9 ± 1.7_a_^a^	81.1 ± 1.8_a_^a^
*Azadirachta indica* bark	51.6 ± 1.2_c_^b^	54.4 ± 1.4_c_^ab^	57.7 ± 1.3_c_^a^	58.4 ± 1.7_c_^a^
*Terminalia arjuna* bark	37.2 ± 1.1_d_^c^	47.3 ± 1.3_c_^b^	61.6 ± 1.3_c_^a^	65.9 ± 1.4_c_^a^
*Ficus religiosa* fruit	55.9 ± 1.4_c_^b^	62.9 ± 1.7_b_^a^	56.2 ± 1.4_c_^b^	63.8 ± 1.6_c_^a^
*Aloe barbadensis* leaves	72.9 ± 1.5_b_^ab^	77.6 ± 1.9_a_^a^	68.0 ± 1.3_b_^b^	71.9 ± 1.2_b_^ab^

Values (mean ± SD) are average of three samples of each medicinal plant material, analyzed individually in triplicate (*n* = 1x3 x 3), (*P* < 0.05); DW= dry weight; Superscript letters within the same row indicate significant (*P*< 0.05) differences of means within the extracting solvent; Subscript letters within the same column indicate significant (*P*< 0.05) differences of means within the plant materials.

The extracts of all the tested medicinal plant materials possessed free radical scavenging properties, but to varying degrees, ranging from 37.2 to 86.6% DPPH^.^ scavenging. Using the shaker extraction technique, generally aq. EE and aq. ME showed better DPPH^.^ scavenging activity. A maximum scavenging activity was offered by aq. EE of *Acacia nilotica* bark (86.6 %), followed by aq. ME of *Moringa oleifera* leaves (86.3%), aq. ME of *Aloe barbadensis* leaves (80.1%), aq. EE *Terminalia arjuna* bark (67.4%)*,* aq. ME of *Ficus religiosa* fruit (63.4%), aq. ME of *Moringa oleifera* root (62.9%)*,* aq. EE of *Azadirachta indica* bark (60.8%)*,* and aq. EE of *Eugenia jambolana* bark (53.9%). The DPPH^. ^scavenging activity seen in the different barks investigated in the present study was found to be lower than that of pine bark (95.1%) [17], while methanolic extract of *Aloe barbadensis* presented DPPH^. ^scavenging activity (72.19%) comparable with that of earlier findings of Hu *et al*. [11].

The ethanolic extracts of roots of *Moringa oleifera,* leaves of *Aloe barbadensis*, and fruit of *Ficus religiosa* prepared by the reflux technique showed better scavenging activity as compared with those of prepared by the shaking technique. These results are in good agreement with the previous findings of Dutra *et al*. [23] who found that among different extraction techniques (reflux, maceration, ultrasound, heating plate) used, extraction made under reflux using ethanol/water (70:30, v/v) exhibited highest DPPH^.^ scavenging activity. The rest of the medicinal plant materials extracts, prepared using shaker extracting method exhibited better scavenging activity than their corresponding extracts, obtained by reflux technique. It has well established that free radical scavenging activity of plant extracts is mainly due to phenolic compounds. This reduction in the radical scavenging activity of the extracts, obtained by the reflux technique might be ascribed to the thermal decomposition of phenolics [23].

### Effects of extracting solvent/technique on the percent inhibition of linoleic acid peroxidation of different plant materials

Inhibition of linoleic acid oxidation determined for extracts of different plant materials as affected by extracting schemes are shown in Table 6. 

**Table 6:** Effects of extracting solvent/technique on the percent inhibition of linoleic acid peroxidation of different medicinal plant materials.

Medicinal plant organs	Extraction by shaker			
	Absolute methanol	Aqueous (80%) methanol	Absolute ethanol	Aqueous (80%) ethanol
*Moringa oleifera* leaves	79.9 ± 2.4_b_^ab^	86.2 ±2.6_a_^a^	73.3 ± 1.5_b_^b^	82.9 ± 1.6_b_^a^
*Moringa oleifera* root	47.6 ± 1.9_e_^b^	66.7 ±2.4_d_^a^	45.3 ± 1.8_e_^b^	65.2 ± 1.3_c_^a^
*Eugenia jambolana* bark	85.2 ± 2.6_a_^ab^	80.4 ± 1.6_b_^b^	90.6 ± 2.7_a_^a^	90.2 ± 1.8_a_^a^
*Acacia nilotica* bark	85.2 ± 1.8_a_^a^	78.2 ± 2.3_b_^b^	86.2 ± 1.6_a_^a^	69.2 ± 2.7_c_^c^
*Azadirachta indica* bark	65.0 ± 2.6_c_^b^	71.4 ± 2.8_c_^a^	47.8 ± 1.8_e_^d^	55.1 ± 1.6_d_^c^
*Terminalia arjuna* bark	31.1 ± 1.2_f_^c^	44.4 ±1.7_e_^c^	61.8 ± 2.4_c_^b^	66.0 ± 2.6_c_^a^
*Ficus religiosa* fruit	59.2 ± 1.7_d_^b^	67.4 ±2.1_d_^a^	54.9 ± 2.1d_d_^c^	60.8 ± 2.4_d_^b^
*Aloe barbadensis* leaves	66.2 ± 1.3_c_^a^	68.3 ±1.4_d_^a^	63.7 ± 1.9_c_^b^	65.9 ± 1.9_c_^ab^
	**Extraction by reflux**			
*Moringa oleifera* leaves	68.2 ± 2.0_b_^b^	82.6 ± 1.6_a_^a^	68.7 ± 2.0_b_^b^	80.5 ± 1.7_b_^a^
*Moringa oleifera* root	40.8 ± 1.6_d_^b^	64.8 ± 2.5_c_^a^	43.2 ± 1.7_d_^b^	63.6 ± 1.9_cd_^a^
*Eugenia jambolana* bark	83.3 ± 2.4_a_^a^	78.1 ± 2.3_a_^b^	88.9 ± 2.6_a_^a^	87.1 ± 1.8_a_^a^
*Acacia nilotica* bark	84.1 ± 1.5_a_^a^	72.1 ± 1.4_b_^b^	85.3 ± 2.5_b_^a^	66.3 ± 1.6_c_^c^
*Azadirachta indica* bark	63.2 ± 1.2_b_^b^	69.1 ± 2.0_bc_^a^	44.3 ± 1.7_d_^d^	50.8 ± 2.0_e_^c^
*Terminalia arjuna* bark	59.4 ± 1.7_c_^a^	36.2 ± 1.4_d_^b^	61.4 ± 1.2_b_^a^	63.2 ± 2.5_cd_^a^
*Ficus religiosa* fruit	59.6 ± 2.3_c_^b^	67.1 ± 2.0_bc_^a^	55.2 ± 1.6_c_^c^	61.9 ± 2.4_d_^b^
*Aloe barbadensis* leaves	64.3 ± 1.9_b_^b^	67.9 ± 1.3_bc_^a^	66.2 ± 1.3_b_^a^	67.3 ± 2.1_c_^a^

Values (mean ± SD) are average of three samples of each medicinal plant material, analyzed individually in triplicate (*n* = 1x3 x 3), (*P* < 0.05); DW= dry weight; Superscript letters within the same row indicate significant (*P*< 0.05) differences of means within the extracting solvent; Subscript letters within the same column indicate significant (*P*< 0.05) differences of means within the plant materials.

The present data were also compared with that of the synthetic antioxidant BHT (reference compound), which exhibited inhibition of linoleic acid oxidation at a level of 81.3 %. Among medicinal plant materials, maximum inhibition was noted by ab. EE of *Eugenia jambolana* bark (90.6%)*,* followed byab. EE *of Acacia nilotica* bark*=* aq. ME of *Moringa oleifera* leaves (86.2%), aq. ME *Azadirachta indica* bark (71.4%)*,* aq. ME of *Aloe barbadensis* leaves (68.3%), aq. ME of *Ficus religiosa* fruits (67.4%), aq. ME of *Moringa oleifera* roots (66.7%)*,* and aq. EE of *Terminalia arjuna* bark (66.0%). The present level of percent inhibition exhibited by *Moringa oleifera* leaf extract (86.2%) was slightly lower than the values (89.7-92.0%) reported by Siddhuraju and Becker [9]. The extents of percent inhibition of linoleic acid (31.1-90.6%) exhibited by tree barks in the present investigation were found to be lower than the values (95.1%) offered by pine bark [17]. The present data revealed that, regardless of the solvent used, the extracts of all medicinal plant materials, prepared using the shaker extracting technique, exhibited higher levels of inhibition of linoleic acid oxidation than those obtained by the reflux method. 

## Conclusions

The results of the present investigation revealed that aqueous solvent (80% methanol, 80% ethanol) extracts of plant materials, prepared by both the shaker and reflux extraction techniques, exhibited better antioxidant activities and higher phenolic contents. Moreover, higher antioxidant extracts yields from the tested plant materials were obtained using the reflux extraction technique than by shaker, regardless of the solvent system used. Contrarily to extraction yield, the total phenolic contents and antioxidant activities of the tested plant materials decreased when these were extracted using the reflux technique. The present data would certainly help to ascertain the potency of the tested medicinal plant materials as potential source of natural antioxidants to be used for nutraceutical and functional food applications. However, further research is needed to identify individual components forming antioxidative system and develop their applications for food and pharmaceutical industries.

## Experimental

### Plant material

The selection of the plant materials in the present study was based on their potential medicinal uses. Medicinal plant parts i.e. barks of *Azadirachta indica* (Neem), *Acacia nilotica* (Desi kiker)*, Eugenia jambolana* (Jaman), *Terminalia arjuna* (Arjun), leaves and roots of *Moringa oleifera* (Sohanjana), fruit of *Ficus religiosa* (Peepal), and leaves of *Aloe barbadensis* (Aloe vera) were collected from plants in the vicinity of the University of Agriculture, Faisalabad, Pakistan. The subject plant material specimens were further authenticated by a taxonomist, Dr. Mansoor Hameed, Department of Botany, University of Agriculture, Faisalabad, Pakistan. 

### Chemicals and reagents

Analytical grade, Merck, Sigma and Fluka brand chemicals and reagents were used for the entire experimentation. 1,1-Diphenyl-2-picrylhydrazyl radical (DPPH*^., ^*Sigma, 90.0 %), linoleic acid, food grade synthetic antioxidant butylated hydroxytoluene (BHT, 99.0 %), Folin-Ciocalteu reagent (2 N), and gallic acid were purchased from Sigma Chemicals Co (St, Louis, MO, USA). All other chemicals (analytical grade) i.e. sodium hydroxide, sodium nitrite, ferrous chloride, ammonium thiocyanate, aluminum chloride, potassium dihydrogen phosphate, dipotassium hydrogen phosphate, used in this study were purchased from Merck (Darmstadt, Germany), unless stated otherwise.

### Extraction of phenolic antioxidants

The air-dried ground (80 mesh) plant material (20 g for each sample) was extracted with each of the solvents – absolute ethanol, absolute methanol, aqueous ethanol (ethanol: water, 80:20 v/v) and aqueous methanol (methanol: water, 80:20 v/v) (200 mL) – for 6 hours at room temperature in an orbital shaker (Gallenkamp, UK), or under reflux on a water bath in separate experiments. The extracts were separated from the residues by filtering through Whatman No. 1 filter paper. The residues were extracted twice with the same fresh solvent and extracts combined. The combined extracts were concentrated and freed of solvent under reduced pressure at 45 °C, using a rotary evaporator (EYELA, SB-651, Rikakikai Co. Ltd. Tokyo, Japan). The dried crude concentrated extracts were weighed to calculate the yield and stored in a refrigerator (- 4 ^o^C), until used for analyses. 

### Evaluation of antioxidant activity of plant materials/extracts

*Determination of total phenolic content (TPC)*: Amount of TP were assessed using the Folin-Ciocalteu reagent [25]. Briefly, the crude extract (50 mg) was mixed with Folin-Ciocalteu reagent (0.5 mL) and deionized water (7.5 mL). The mixture was kept at room temperature for 10 min, and then 20% sodium carbonate (w/v, 1.5 mL) was added. The mixture was heated in a water bath at 40 ^o^C for 20 min and then cooled in an ice bath; absorbance was read at 755 nm using a spectrophotometer (U-2001, Hitachi Instruments Inc., Tokyo, Japan). Amounts of TP were calculated using gallic acid calibration curve within range of 10-100 mgL^-1^(R^2^ = 0.9986). The results were expressed as gallic acid equivalents (GAE) g/100g of dry plant matter. All samples were analyzed thrice and the results averaged. The results are reported on dry weight basis (DW).

*Determination of total flavonoid contents (TFC)*: The TFC were measured following a previously reported spectrophotometric method [26]. Briefly, extracts of each plant material (1 mL containing 0.1 mg/mL) were diluted with water (4 mL) in a 10 mL volumetric flask. Initially, 5% NaNO_2_ solution (0.3 mL) was added to each volumetric flask; at 5 min, 10% AlCl_3_ (0.3 mL) was added; and at 6 min, 1.0 M NaOH (2 mL) was added. Water (2.4 mL) was then added to the reaction flask and mixed well. Absorbance of the reaction mixture was read at 510 nm. TFC were determined as catechin equivalents (g/100g of dry weight). Three readings were taken for each sample and the results averaged.

*Determination of reducing power*: The reducing power of the extracts was determined according to the procedure described earlier [27], with a slight modification. Concentrated extract (2.5-10.0 mg) was mixed with sodium phosphate buffer (5.0 mL, 0.2 M, pH 6.6) and potassium ferricyanide (5.0 mL, 1.0%); the mixture was incubated at 50 ^o^C for 20 min. Then 10% trichloroacetic acid (5 mL) was added and the mixture centrifuged at 980 *g* for 10 min at 5 °C in a refrigerated centrifuge (CHM-17; Kokusan Denki, Tokyo, Japan). The upper layer of the solution (5.0 mL) was decanted and diluted with 5.0 mL of distilled water and ferric chloride (1.0 mL, 0.1%), and absorbance read at 700 nm using a spectrophotometer (U-2001, Hitachi Instruments Inc., Tokyo, Japan). All samples were analyzed thrice and the results averaged.

*DPPH^.^ scavenging assay*: 1, 1^’^–diphenyl–2-picrylhydrazyl (DPPH) free radical scavenging activity of the extracts was assessed using the procedure reported earlier [28]. Briefly, to extract (1.0 mL) containing 25 μg/mL of dry matter in methanol, freshly prepared solution of DPPH (0.025 g/L, 5.0 mL) was added. Absorbance at 0, 0.5, 1, 2, 5 and 10 min was measured at 515 nm using a spectrophotometer. The scavenging amounts of DPPH radical (DPPH^.^) was calculated from a calibration curve. Absorbance read at the 5th min was used for comparison of radical scavenging activity of the extracts. 

*Determination of antioxidant activity in linoleic acid system:* The antioxidant activity of the tested plant extracts was also determined by measuring the oxidation of linoleic acid [28]. Five mg of each plant extract were added separately to a solution of linoleic acid (0.13 mL), 99.8% ethanol (10 mL) and 0.2 M sodium phosphate buffer (pH 7, 10 mL). The mixture was made up to 25 mL with distilled water and incubated at 40 ^o^C up to 360 h. Extent of oxidation was measured by peroxide value applying thiocyanate method as described by Yen *et al*. [27]. Briefly, ethanol (75% v/v, 10 mL ), aqueous solution of ammonium thiocyanate (30% w/v, 0.2 mL), sample solution (0.2 mL) and ferrous chloride (FeCl_2_) solution (20 mM in 3.5% HCl; v/v, 0.2 mL) were added sequentially. After 3 min of stirring, the absorption was measured at 500 nm using a spectrophotometer (U-2001, Hitachi Instruments Inc., Tokyo, Japan). A control contained all reagents with exception of extracts. Synthetic antioxidants butylated hydroxytoluene (BHT) was used as a positive control. Percent inhibition of linoleic acid oxidation was calculated with the following equation: 100 – [(increase in absorbance of sample at 360 h / increase in absorbance of control at 360 h) × 100], to express antioxidant activity.

### Statistical analysis

Three samples of each plant material were assayed. Each sample was analyzed individually in triplicate and data are reported as mean (n = 3 x 3 x1) ± SD (n = 3 x 3 x 1). Data were analyzed using a 2- way analysis of variance (ANOVA) using Minitab 2000 Version 13.2 statistical software (Minitab Inc. Pennsylvania, USA). A probability value of *p* ≤ 0.05 was considered to denote a statistically significance difference. 
